# Comparison of the Polyamine Content of Five Spring Flowers with Wheat Germ as a Rich Anti-aging Polyamine Source for Preparation of Nutraceutical Products

**DOI:** 10.5812/ijpr-134938

**Published:** 2023-04-25

**Authors:** Maryam Mohajeri, Seyed Abdulmajid Ayatollahi, Mohammad Goli, Shaya Mokhtari, Maryam Khandan, Azadeh Nasiri, Farzad Kobarfard

**Affiliations:** 1 Phytochemistry Research Center, Shahid Beheshti University of Medical Sciences, Tehran, Iran; 2 Department of Pharmacognosy, School of Pharmacy, Shahid Beheshti University of Medical Sciences, Tehran, Iran; 3 Department of Food Science and Technology, Laser and Biophotonics in Biotechnologies Research center, Isfahan (Khorasgan) Branch, Islamic Azad University, Isfahan, Iran; 4 Central Research Laboratories, Shahid Beheshti University of Medical Sciences, Tehran, Iran; 5 Vice-Chancellor for Food and Drug Affairs, Shahid Beheshti University of Medical Science, Tehran, Iran; 6 Department of Medicinal Chemistry, School of Pharmacy, Shahid Beheshti University of Medical Sciences, Tehran, Iran

**Keywords:** Polyamines, Spermidine, Spring Flowers, LC-MS/MS

## Abstract

Polyamines prolong longevity due to their role in cell proliferation and are regarded as an essential group of anti-aging substances that reduce the risk of cardiovascular, neurological, and chronic inflammatory illnesses, as well as cancer. Because of its importance in growth and tissue regeneration, discovering polyamine-rich sources has gotten a lot of interest. Given the role of polyamines in controlling plant growth and physiological changes in the spring after cold winter stress, high polyamine concentrations in quickly growing plant tissues such as flowers, blossoms, and germs are possible. Based on this premise, five different spring flowers were selected and isolated from relevant plants, dried, and then quantified for the first time using an accurate, simple, and repeatable quantification method, liquid chromatography–tandem mass spectrometry. According to the amount of spermidine found in the samples investigated in this study, dried flower powders of *Wisteria sinensis* (244.18 µg/g), *Lonicera caprifolium* (217.28 µg/g), and *Jasminum officinale* (200.33 µg/g) appear to be a good source of spermidine. With additional research, *W. sinensis* dried flower powder is a good source of polyamines, whereas *L. caprifolium* and *J. officinale* dried flower powders are recommended as a rich source of spermidine for the preparation of natural supplements for people over the age of 30 to improve cell proliferation and anti-aging.

## 1. Background

Polyamines (PAs) are aliphatic molecules with two or more amine groups with a low molecular weight. PA biosynthesis involves metabolism, catabolism, and transfer, all done by different enzymes. Aging reduces the activity of these enzymes and, as a result, the synthesis of PAs (1-3), which reduces cellular proliferation and hence promotes aging (4, 5). High PA levels in the body promote skin regeneration and hair and nail growth and prevent physiological aging (6, 7). Oral PAs consumed daily maintain a high blood level and stimulate cell proliferation (6, 8). Putrescine (Put), spermidine (Spd), and spermine (Spm) are three of the most abundant natural PAs discovered in the human body (1). PAs are growth regulators and/or plant stimulators because they help with cell proliferation and differentiation, resulting in various physiological processes, such as stress tolerance and flower induction (9). Many studies sought to determine the levels of polyamines in various foods and plant tissues as polyamine sources. These investigations show polyamine levels are highest in fast-growing tissues such as germs, spring flowers, and blooms (10, 11). Plant polyamine levels are affected by seasonal and temperature fluctuations (12). Polyamine levels in plants reach their lowest levels during cold winters, resulting in bud dormancy (12). The quality and quantity of flowering fluctuate due to the cold stress tolerance mechanism, depending on the duration of bud hibernation and the degree of coldness (13). As a result, the confluence of rapid spring physiological changes and the previously discussed stress was expected to be connected with polyamine increase in plants. Herein, we examined the amounts of PAs in five different species of spring flowers: *Robinia pseudoacacia* and *Wisteria sinensis* in the Fabaceae (leguminosea) family, *Jasminum officinale *and *Jasminum polyanthum* in the Oliveaceae family, and *Lonicera caprifolium* in the Caprifoliaceae family.

*Robinia pseudoacacia*, also known as black locust (or false acacia), is a medium-sized hardwood deciduous tree in the Fabaceae family’s tribe *Robinieae*. It is commonly grown in North America, Europe, Southern Africa, and Asia (14). *Robinia pseudoacacia* flowers, bark, and leaves are used in traditional medicine for antitussive, laxative, and cholagogue purposes (15, 16). *Wisteria sinensis*, sometimes known as Chinese *Wisteria*, is a flowering plant in the Fabaceae family. It is a deciduous vine that grows to 20 - 30 meters (66 - 98 feet) and is extensively cultivated in the spring (15). *W. sinensis* leaves and flowers are also used as tea replacements (17). Many eastern pharmacists employ *Wisteria* gall extracts to treat patients with gastric cancer, breast cancer, and stomach cancer, as well as rheumatoid arthritis (18, 19). *Jasminum* is a genus of shrubs and vines from the olive family (Oleaceae). Because of their distinctive floral scent, jasmines are frequently grown. This plant’s flowers can be found in *J. officinale* species like *polyanthum* and others (16). *Jasminum* flowers are used to treat eye problems, boils, vesicles, ulcers, and skin conditions. Breast tumors, aphthous, stomatitis, toothache, and ulcers in the mouth, throat, and gums can all be treated with leaf extract (20). Its leaves were formerly ground into juice and used as a sedative, mild anesthetic, and astringent to treat urinary tract infections (21). A species of perennial flowering plant belonging to *Lonicera* and the family Caprifoliaceae is called *L. caprifolium*. It is indigenous to portions of North America, South East Asia, and Europe. Its leaves can be used to distinguish it from *L. periclymenum*, the most prevalent species in Europe. The plant’s herbaceous parts can be a diuretic (17, 22).

Comprehensive investigations of several polyamine measuring techniques were conducted (23-26). Since polyamines lack a chromophore in their structure, analytical techniques based on UV absorption and fluorescent detection would not be appropriate. Therefore, the polyamine analysis can be performed without using mass spectrometric (MS) detectors or with appropriate derivatizing agents by UV or fluorescent detectors.

High-pressure liquid chromatography (HPLC) coupled to tandem mass spectrometry (MS/MS) analyzers is chosen over conventional detection methods because it eliminates interference problems (18-21, 23, 24, 27-32). Due to strong acids in non-derivatized PAs analytical methods, column performance degraded (18, 19, 21, 23, 25). Therefore, derivatization is favored in polyamine detection with MS/MS analyzers since it lowers interference and boosts repeatability (20). We provided a simple, shortened, and isocratic analysis that took advantage of derivatization techniques while avoiding the use of potent acids and typical interferences seen in classical LC-MS techniques (33).

## 2. Objectives

To the authors’ knowledge, no research has been conducted on estimating the PA levels in spring flowers. We focused on fast-growing plant tissues, namely flowers, and blooms, as prospective candidates while looking for plant tissues that contained high concentrations of Pas (10, 11). This study’s primary objective was to examine the quantities of free PAs (Put, Spd, and Spm) in four genera of spring flowers, including *L. caprifolium*, *Wisteria sinensis*, *R. pseudoacacia*, *J. polyanthum*, and *J. officinale*.

## 3. Methods

### 3.1. Plant Material

The spring flowers planted in a commercial orchard in Tehran City, Tehran Province, Iran, were the subject of this study. *R. pseudoacacia*, *W. sinensis*, *L. caprifolium*, *J. officinale*, and *J. polyanthum* were verified by the Department of Botany, School of Pharmacy, Shahid Beheshti University of Medical Sciences, with the Voucher specimen numbers of HSP-106, HSP-107, HSP-110, HSP-108, and HSP-109 respectively. *R. pseudoacacia* was gathered between 22 and 30 May 2022, *W. sinensis* between 1 and 7 April 2022, *L. caprifolium* between 1 and 7 May 2022, and *J. officinale* and *J. polyanthum* between 23 April and 7 May. The Shahid Beheshti Faculty of Pharmacy’s medicinal plant laboratory identified the flowers. After being detached from the leaves and stems, they were dried in a dark area at room temperature. The dried flowers were then milled into a fine powder. They were used for further experiments.

### 3.2. Reagents and Standards

Sigma-Aldrich provided the Spd, Put, and Spm standards, 1, 6-diaminohexan (internal standard), and isobutyl chloroformate (IBCF) (Saint Quentin-Fallavier, France). HPLC-grade ethanol and acetonitrile were purchased from Merck. All chemical reagents (disodium hydrogen phosphate and sodium hydroxide) were of analytical grade and were used without further purification.

### 3.3. Sample Preparation

The dried flower powders (2 g) were combined with 8 mL of ethanol: water solution (2:3 V/V) for 1 h at RT. After that, the mixture was centrifuged for 20 min at 12,000 rpm, with the supernatant being discarded. There were two rounds of washing. The plant residue was mixed with 8 mL of water and agitated at 300 rpm for 1 h at RT. The mixture was stirred while citric acid (anhydrous) was gradually added until the pH was adjusted to 4.0 for 2 h. After centrifuging the mixture for 20 min at 12000 rpm, the supernatant was evaporated using a rotary evaporator to produce a dry powder. The derivatization process was applied to the obtained dry powder.

Derivatization and analysis were carried out in accordance with our earlier work (33). Briefly, a 15 mL falcon tube containing 2 mL of toluene, 1 mL of phosphate buffer (0.5 M, pH 12), and 100 μL of IBCF was filled with 1 mL of the sample solution (100 mg/mL water) and 10 μL of 1, 6 diaminohexane solution (10 mg/mL water). The organic phase was separated from the aqueous phase after 10 min vortex shaking at room temperature, dried using anhydrous Na_2_SO_4_ (2g), and then centrifuged (10 min at 3500 rpm). Using an alkaline methanol and NaOH (5M) solution, excess IBCF was eliminated from the supernatant solution. Then, 100 μL of the toluene layer was dried completely under a nitrogen gas stream. Eventually, the residue was dissolved in 1 mL of acetonitrile to be used for analysis with the LC–MS/MS. 

### 3.4. LC-MS/MS Analysis

MS/MS analysis was performed on an HPLC separation module (Agilent series, 1200, Germany) equipped with a quaternary solvent delivery system, degasser, autosampler, and column heater, coupled with a Triple Quadrupole LC–MS (Agilent Technologies, 6410, California, USA). An MZ Analysis C18 column (150 mm, 4.6 mm, and particle size: 5 μm) was used for separation. The mobile phase for elution was established in an isocratic mode; water acidified with 0.1% formic acid (FA) (as phase A) and acetonitrile acidified with 0.1% formic acid (as phase B) at a flow rate of 0.35 mL /min. 18.5% A/81.5% B in an isocratic eluent was used for the separation. Each sample was injected in 5 µL. The autosampler temperature was 25°C, while the column temperature was set at 35°C. The following conditions were used throughout the analysis: The following parameters were used for mass spectrometry: spray voltage of 5.0 kV, capillary temperature of 320°C, gas flow rate of 8 L/min, and capillary voltage of 32 V. All analytes were found in positive mode during measurements of multiple reaction monitoring (MRM). Table 1 lists the analyte’s ideal analytical conditions (33).

**Table 1.** MRM Acquisition Settings of the IBCF Polyamines and Internal Standard

Compound Name	Number of IBCF Groups	MW	MRM Transition (M/Z)	Normalized CE	Fragmentor	Retention Time (Min)
**Put**	2	288.2	288.2 > 115.1	10	20	6.5
			288.2 > 215.1	5	20	6.5
**Spd**	3	445.2	446.2 > 372	10	100	8.5
			446.2 > 298	10	100	8.5
**Spm**	4	602.66	603.7 > 529.4	10	100	13
			603.7 > 455.3	20	100	13
**1,6-Diaminohexane**	2	316.2	317.2 > 243.1	5	100	7.5
			317.2 > 143.1	15	100	7.5

Abbreviations: Put, putrescine; Spd, spermidine; Spm, spermine.

### 3.5. Method Validation

A linearity test, as well as calculations of inter- and intra-run precision, accuracy, and limit of quantification (LOQ), were used to validate the approach. To create a calibration curve, a 10 mg/ mL polyamine stock solution was prepared. Working standard solutions at the concentration levels of 0.2, 0.4, 0.6, 0.8, and 1 mg/mL for spermidine and 0.05, 0.1, 0.2, 0.4, 0.6, 0.8, and 1 mg/ mL for spermine and putrescine were prepared. 10 μL of 1, 6-diaminohexan (internal standard) solution (10 mg/ mL in 0.1 M HCl) was added to 1 mL of each standard sample. The calibration curves were constructed by plotting peak area ratios (compound peak area/ internal standard peak area) against the standard concentrations. The calibration curves’ correlation coefficient (R^2^) was used to assess the linearity. Using quality control (QC) samples of each polyamine at 3 - 5 various concentrations, accuracy and precision were evaluated. Precision was measured as the relative standard deviation (%RSD), while accuracy was calculated as the relative error (%bias). While inter-day validation required sample analysis on three different days, intra-day validation was confirmed by three examinations of samples on the same day. The lowest quantifiable concentration with a signal-to-noise ratio of more than 10 was known as the LOQ. By adding a specific amount of standard solution (100 µL of the standards (10 mg/mL) to 0.1 g of enriched powder and by adding distilled water diluted to 1 mL) to the samples and using the following equation, the matrix effect was assessed:


Equation 1. $$Matrix\;effect\;\left(\%\right)=\left[\frac{\left(A-B\right)}{B}\right]\times 100$$

Where A and B are as follows:


Equation 2. $$A=\frac{Spiked\;solution\;peak\;area}{IS\;peak\;area}$$

Equation 3. $$A=\frac{Standard\;solution\;peak\;area}{IS\;peak\;area}$$

### 3.6. Statistical Analysis

All tests were carried out using statistical SPSS software version 16 in a totally random order (Chicago, SPSS Inc., USA). The weight of polyamine to the weight of dried wheat germ powder was a determining factor. In each type of treatment, the polyamine weight ratio (Z%) of the three polyamines (Put, Spd, and Spm) served as a determining factor. Duncan’s multiple range test revealed significant differences (P < 0.05) at the 95% confidential level.

## 4. Results

### 4.1. Liquid Chromatography-tandem Mass Spectrometry

In order to identify polyamine isolated from spring flowers, the current study proposes a straightforward isocratic approach based on derivatization with isobutyl chloroformate on a reverse phase chromatographic column (C18) utilizing an Agilent HP series 1100 binary pump LC-MS/MS system. The residue is reconstituted in acetonitrile and injected into the LC-MS after drying the derivatized sample. The polyamines were detected in the positive ESI mode and by multiple reactions monitoring (MRM) technique. Direct injection of the standard solutions into the mass spectrometer using a syringe pump at a flow rate of 0.35 mL/min was used to optimize the precursor ions, product ions, fragmentor voltage (Fr), and collision energy (CE). The [M + H] + ions were regarded as precursor ions in the MRM study. The [M + H - OCH2C3H7] + ion appeared as the basis peak for quantifying all derivatized polyamines. The most intense peaks in the product ion spectra of carbamylated polyamines are ion fragments of 298 and 372 (m/z) for spermidine, 115 and 215 (m/z) for putrescine, and 455 and 529 (m/z) for spermine. 1, 6-diaminohexane was employed as the internal standard (IS) to measure polyamines and eliminate the matrix effect (Table 1). The observed matrix effect during the analysis of all five flower extracts was less than wheat germ. Additionally, the separation phase of the wheat germ production process is not necessary for these flowers. The advantages of these flowers support the claim that they may provide a good source of polyamines, similar to wheat germ. It was discovered that the extract of spring flowers has a significantly reduced matrix effect in HPLC polyamine analysis compared to the solution of PAs extracted from the wheat germ (Table 2).

**Table 2.** Calibration Range, Linear Regression Equation, Limits of Quantification (LOQ), Matrix Effect, and Recovery of Polyamines

Analytes	Calibration Rang (mg/mL)	Linear Regression Equation	R^2^	LOQ (mg/mL)	Matrix Effect (%)
**Putrescine**	0.1 - 1	Y = 1.6433x + 0.2874	0.9957	0.1	10.65
**Spermidine**	0.2 - 1	Y = 1.3317x + 0.3855	0.9982	0.2	2.5
**Spermine**	0.05 - 1	Y = 3.0004x + 0.0714	0.9979	0.05	5.1

### 4.2. Method Validation

To confirm the linearity of calibration curves, ratios of analyte peak area to internal standard peak area in the range of 0.05 - 1 mg/mL were plotted versus polyamine concentration. Three or four independent replicates of each of the seven concentration levels were performed on three different days. For all analytes, the regression equations were determined to be linear with an appropriate correlation coefficient, R^2^ > 0.99. Table 2 displays the results. The developed method was validated by evaluating the accuracy and precision of quality control (QC) samples at 3 - 5 concentrations, as shown in Table 3. The relative standard deviation (%RSD) was used to assess precision, while the relative error rate (%bias) was used to assess accuracy. The validity of the LC-MS/MS method was established because accuracy and precision values were less than 20%. The limit of quantification (LOQ) for spermidine was set at 0.2 mg/mL, putrescine at 0.05 mg/mL, and spermine at 0.1 mg/mL (Table 2).

Matrix effects ranging from 10% to 19% were computed for all three analytes (Table 2). The relative standard deviation (%RSD) was used to assess precision, while the relative error rate (%bias) was used to assess accuracy. The intra-day (n = 3) precision (%RSD) and accuracy (%bias) for spermidine were 1.5 - 4.5% and 95.5 - 105.5%, respectively, for spermine, 4.32 - 19.5% and 80.5 - 111.7%, and 2.425 - 8.27% and 91.73 - 97.58% for putrescine. However, the inter-day (n = 3) precision and accuracy for spermidine were 4.47 - 9.5% and 103.2 - 112.4%, 2.64 - 16.23% and 85.9 - 116.2% for spermine, and 7.135 - 10.53% and 89.47 - 97.86% for putrescine (Table 3).

**Table 3.** Intra-day and Inter-day Validation of Polyamines

QC Concentration (mg/mL)	Intra-day (n = 3)		Inter-day (n = 3)	
	Accuracy (%Bias)	Precision (%RSD)	Accuracy (%Bias)	Precision (%RSD)
**Put**				
0.05	97.58	2.425	91.63	8.37
0.2	91.73	7.04	92.86	7.135
0.4	92.96	8.27	89.47	10.53
**Spd**				
0.2	97.3	3.5	109.2	9.5
0.4	105.8	1.5	112.4	4.47
0.6	95.5	4.5	103.2	7.3
0.8	98.9	1.12	103.7	4.8
**Spm**				
0.1	108.2	8.23	113.13	13.15
0.2	111.7	11.69	116.2	16.23
0.4	104.3	4.32	104.06	3.79
0.6	93.9	6.1	99.49	2.64
0.8	88.6	11.5	95.9	4.14
1	80.5	19.5	85.9	14.09

Abbreviations: Put, putrescine; Spd, spermidine; Spm, spermine.

### 4.3. Polyamine Concentrations in Spring Flowers Samples

**Figure 1.**
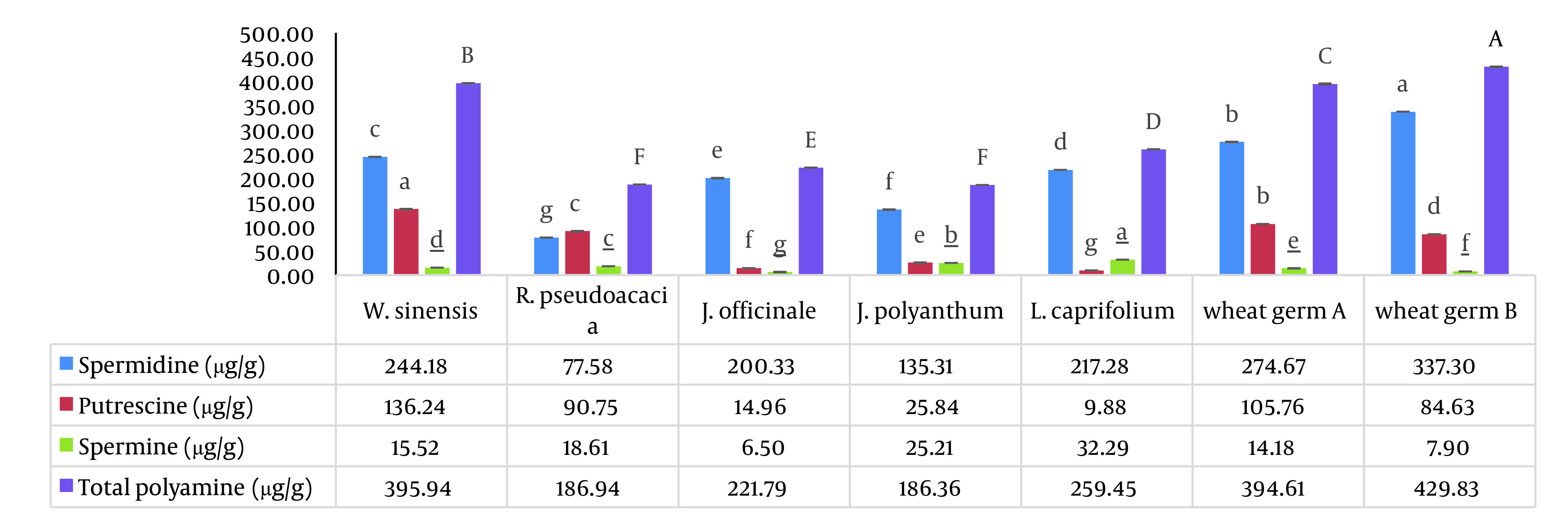
The statistical comparison of the concentration of each polyamine in seven treatments separately. Mean ± SD values (n = 3), followed by the same letter on columns for each treatment (each polyamine), have no significant differences in P ≤ .05 by ANOVA.

**Figure 2.**
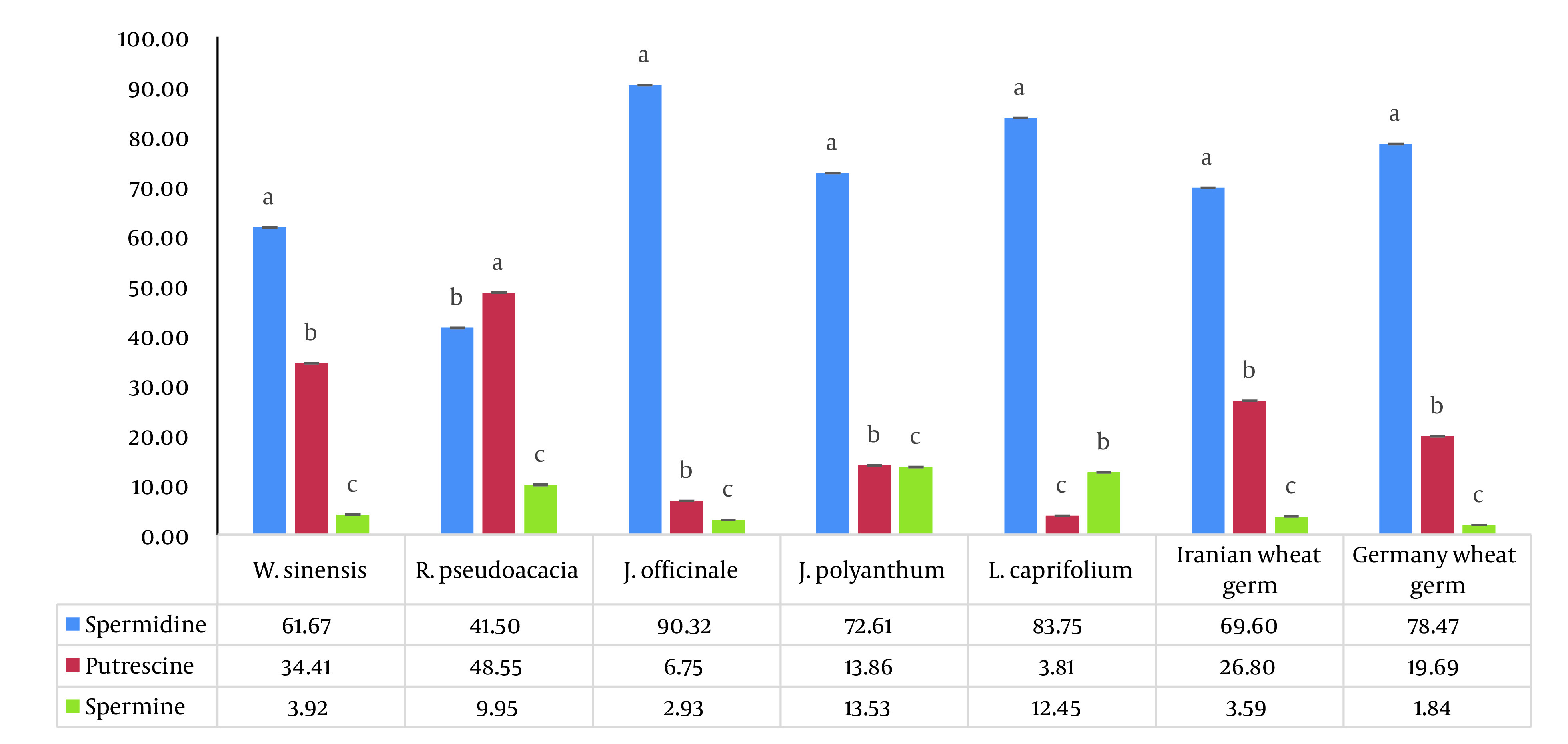
The statistical comparison of Z % of all three polyamines (putrescine, spermidine, and spermine) in each type of flower separately. Mean ± SD values (n = 3), followed by the same letter on columns for each treatment (each flower), have no significant differences in P ≤ 0.05 by ANOVA.

Here, we examined the concentrations of free PAs (Put, Spm, and Spd) in five spring flowers: *R. pseudoacacia*, *W. sinensis*, *J. officinale*, *J. polyanthum*, and *L. caprifolium*. The weight ratio of PAs to the weight of dried flower powder in five spring flowers is shown in Figure 1 in order to compare the polyamine concentrations. Additionally, Figure 2 provides the proportion of each polyamine’s weight in percent to the sum of all polyamines’ weight in the dried flower powders (Z %). Index (Z), which we defined as the fraction blow, was created to compare the proportion of a particular polyamine across all the PAs that were investigated.


Equation 4. $$Z=\frac{Wt\;ratio\;of\;each\;polyamine\;}{Wt\;ratio\;of\;Put+Spm\;+\;Spd}\times 100$$

## 5. Discussion

PAs are important in various physiological processes such as cell division and elongation, flower formation, leaf senescence delay, fruit ripening, and pollen grain (34). According to the literature study compiled in Table 4 (23-26, 34), cereals contain the highest amount of Spd, and fruits contain the highest amount of Put among the various plant types(2). There were also some investigations that polyamine quantities are at their highest in fast-growing tissues, including germination, spring blooms, and flowers (10, 23, 35). Malik and Bradford (10) investigated different flower-induced conditions that elicit different responses to free polyamine levels in olive (Olea europaea) leaves. Free polyamine concentrations were examined by Liu and Moriguchi (36) during four distinct stages of peach (Prunus persica L. Batsch cv. Akatsuki) flower development. They demonstrated that among the free PAs, Spd had the highest concentration throughout flower development (36). According to Naseri et al., seasonal and temperature changes affect polyamine levels in almond spring buds and blooms (12). Additionally, it was noted that during the cold winter months, when plant polyamine levels are at their lowest, bud dormancy occurs (37). The degree of cold and the length of bud hibernation affect flowering quality and quantity (13). The research above predicted that spring flowers would have a higher concentration of PAs than other flowers. Here, we examined the concentrations of free PAs (Put, Spm, and Spd) in five spring flowers: *R. pseudoacacia, W. sinensis, J. officinale, J. polyanthum, and L. caprifolium*.

**Table 4.** Comparison of Polyamine Content in Various Plants

No.	Extraction from	Put (µg/g)	Spd (µg/g)	Spm (µg/g)	References
**1**	Fruits	nd - 136.98	1.009 - 14.33	nd - 5.06	(2)
**2**	Vegetables	0.5 - 69.99	1.008 - 58.18	nd - 10.93	(2)
**3**	Legumes and soybean products	nd - 46.28	0.146 - 208.33	nd - 68.99	(2)
**4**	Nuts and oilseeds	2.99 - 43	5.99 - 55.99	12.75 - 33.39	(2)
**5**	Cereals-rice, wheat germ	0.2 - 62	0.409 - 356.3	nd - 146.09	(2)
**6**	*Cucumis satius* (cucumber) root	0.15	0.41	0.05	(35)
**7**	*Cucumis satius* (cucumber)stem	0.06	0.2	0.06	(35)
**8**	*Cucumis satius* (cucumber) petiol	0.01	0.18	0.08	(35)
**9**	*Cucumis satius* (cucumber) calyx	0.04	0.41	0.22	(35)
**10**	Peach tree bloom	8.72	34.52	20.39	(38)
**11**	Cauliflower	3.08 - 4.49	21.78 - 27.89	9.71 - 12.95	(23)
**12**	Green tea leaf	15.25	43.27	35.81	(23)
**13**	Black tea leaf	2.2	13.01	23.07	(23)
**14**	*Arabidopsis thaliana* root	-	6.09	4.2	(24)
**15**	*Arabidopsis thaliana* leaf	-	8.41	3.099	(24)
**16**	*Arabidopsis thaliana* flower	-	49.99	23.9	(24)
**17**	*Senecio riddellii* (stem)	0.01	0.04	0.06	(25)
**18**	*Senecio riddellii* (leaves)	0.05	0.07	0.11	(25)
**19**	*Senecio riddellii* (root)	0.049	0.02	0.02	(25)
**20**	*Crotalaria retusa* (stem)	0.01	0.10	0.09	(25)
**21**	*Crotalaria retusa* (leaf)	0.01	0.21	0.07	(25)
**22**	*Crotalaria retusa* (root)	0.03	0.40	0.05	(25)
**23**	Tobacco (*Nicotiana tabacum*)	22.21	80.41	10.32	(23)
**24**	*Amaranthus cruentus*	3.79	42.5	9.5	(29)
**25**	*Amaranthus hypochondriacus*	2.03	53.07	10.32	(29)
**26**	*Avena sativa* (oat)	3.6	21.49	4.25	(29)
**27**	*Lemna gibba* (duckweed) leaf	0.62	83.33	8.29	(29)
**28**	*Pharbitis nil* (Japanese morning glory)	-	59.79	-	(29)
**29**	Kinnow mandarin trees (leaf)	8.27 - 14.28	10.35 - 22.61	4.73 - 7.68	(26)
**30**	Kinnow mandarin trees (stem)	49.96 - 81.69	7.19 - 19.16	2.48 - 5.61	(26)
**31**	Olive (*Olea europaea*) leaf	3.35	3.92	1.92	(10)
**32**	Olive (*Olea europaea*) flower	11.46	12.64	6.68	(10)
**33**	Olive (*Olea europaea*) fruit	3.61	3.92	2.22	(10)
**34**	*Rosa damascena*	0.22	0.41	0.14	(34)

The aforementioned flowers had high levels of polyamines, particularly Spd. With the exception of R. *pseudoacacia*, all five flowers had greater Spd than other PAs. Bush flowers had higher Spd concentrations (200 – 244 µg/g), whereas *R. pseudoacacia* trees had higher Put concentrations (90.75 µg/g). In general, the amount of Spd in the *W. sinensis* flower was higher than that in other flowers (244.15 µg/g). According to the results given by Fujihara and Yoneyama, it may be explained by the fact that the calyx size of *W. sinensis* was greater than the same tissue in *J. officinale*, *J. polyanthum*, and *L. caprifolium* (39). All five floral powders contained no more than 32.27 µg/g of spm, which is consistent with the findings of the published literature (2).

According to Z% comparisons, Spm had the lowest proportion (3 – 13%), whereas Put, Spd, and Spm had the highest proportions (49%, 90%, and 13%) in *R. pseudoacacia, J. officinale, and J. polyanthum*, respectively (Figure 2). The results obtained supported the notion that *J. officinale* is a reliable source of Spd. *Jasminum officinale* had less matrix effect than wheat germ, a significant source of Spd; extracting Spd from this flower did not necessitate physical separation. According to this study, the amount of spermidine in *W. sinensis* is comparable to two different sources of wheat germ as a rich source of spermidine (Figure 1). In comparison to two different sources of wheat germ, *W. sinensis* may be a good source of Put and Spd, whereas *J. officinale* and *L. caprifolium* flowers may be good sources of Spd (Figures 1 and 2). Of course, additional research is needed to support our findings. The most significant part of our discovery is that among the examined spring blooms and flowers, such high Spd values have never, to the best of our knowledge, been reported (Table 3). To the best of our knowledge, these plants’ first polyamines have been measured, and the amounts discovered were very high. It confirmed our initial hypotheses that quickly growing tissues that previously experienced cold stress would be rich in PAs, particularly Spd.

### 5.1. Conclusions

This paper is the first on the amounts of polyamine found in five different types of spring flowers (*R. pseudoacacia*, *W. sinensis*, *J. officinale, J. polyanthum*, and *L. caprifolium*). It’s interesting to note that the measured spermidine levels were equivalent to the spermidine content of wheat germ, a well-known food source. Comparing the polyamine concentrations in these flowers revealed that *W. sinensis* (244.15 µg/g) and *R. pseudoacacia* (77.5 µg/g) had the highest and lowest amounts of Spd, respectively. In particular, Spd had substantial polyamine concentrations in *W. sinensis*, *J. officinale*, and *L. caprifolium*. The three species of *J. officinale*, *R. pseudoacacia*, and *J. polyanthum* with the highest Z % were Spd (90%), Put (49%), and Spm (13%), respectively. Thus, the data obtained support the notion that these flowers are good sources of Spd, similar to wheat germ as a typical source of polyamines. Additionally, the separating step necessary for wheat germ does not need to be done when using spring flowers as a polyamine source. It is important to note that the previously created method for determining the presence of polyamines in wheat germ was used successfully in a sample of five spring flowers with a reduced matrix effect. Considering that all of the spring flowers covered in this study are used in conventional therapy, in consideration of additional research, *W. sinensis, J. officinale, J. polyanthum, *and* L. caprifolium*, along with wheat germ, can be recommended as healthy food sources for those over 30 who want to improve cell proliferation and anti-aging. For the production of natural supplements, *J. officinale* and *L. caprifolium* can also be regarded as rich sources of spermidine (better than wheat germ).

## References

[1] Agostinelli E, Marques MP, Calheiros R, Gil FP, Tempera G, Viceconte N (2010). Polyamines: fundamental characters in chemistry and biology.. Amino Acids..

[2] Munoz-Esparza NC, Latorre-Moratalla ML, Comas-Baste O, Toro-Funes N, Veciana-Nogues MT, Vidal-Carou MC (2019). Polyamines in Food.. Front Nutr..

[3] Gomez-Gallego C, Kumar H, Garcia-Mantrana I, du Toit E, Suomela JP, Linderborg KM (2017). Breast milk polyamines and microbiota interactions: Impact of mode of delivery and geographical location.. Ann Nutr Metab..

[4] Yoshinaga K, Ishizuka J, Evers BM, Townsend CM, Thompson JC (1993). Age-related changes in polyamine biosynthesis after fasting and refeeding.. Exp Gerontol..

[5] Soda K, Kano Y, Sakuragi M, Takao K, Lefor A, Konishi F (2009). Long-term oral polyamine intake increases blood polyamine concentrations.. J Nutr Sci Vitaminol (Tokyo)..

[6] Tong D, Hill JA (2017). Spermidine promotes cardioprotective autophagy.. Circ Res..

[7] Calandra RS, Rulli SB, Frungieri MB, Suescun MO, González-Calvar SI (1996). Polyamines in the male reproductive system.. Acta Physiol Pharmacol Ther Latinoam..

[8] Madeo F, Eisenberg T, Pietrocola F, Kroemer G (2018). Spermidine in health and disease.. Science..

[9] Pritsa TS, Voyiatzis DG (2004). Seasonal changes in polyamine content of vegetative and reproductive olive organs in relation to floral initiation, anthesis, and fruit development.. Aust J Agric Res..

[10] Malik NSA, Bradford JM (2007). Different flower-inducing conditions elicit different responses for free polyamine levels in olive (<i>olea europaea</i>) leaves.. J Jpn Soc Hortic Sci ..

[11] Bagheri S, Abedy B, Rahemi M, Nemati H, Rowshan V (2017). Changes in polyamine levels in relationship to the growth and development of parthenocarpic fruits (shotberries) of olive (Olea europaea L.).. Sci Hortic..

[12] Naseri S, Gholami M, Baninasab B (2019). Changes in polyamines during bud dormancy in almond cultivars differing in their flowering date.. Sci Hortic..

[13] Wang SY, Faust M (1994). Changes in polyamine content during dormancy in flower buds of `Anna' apple.. J Am Soc Hortic Sci ..

[14] Atha DE, Forrest T, Naczi RFC, Pace MC, Rubin M, Schuler JA (2016). The historic and extant spontaneous vascular flora of The New York Botanical Garden.. Brittonia..

[15] Kim HS, Jang JM, Yun SY, Zhou D, Piao Y, Ha HC (2019). Effect of robinia pseudoacacia leaf extract on interleukin-1beta-mediated tumor angiogenesis.. In Vivo..

[16] Leporatti ML, Ivancheva S (2003). Preliminary comparative analysis of medicinal plants used in the traditional medicine of Bulgaria and Italy.. J Ethnopharmacol..

[17] Mohamed MA, Hamed MM, Abdou AM, Ahmed WS, Saad AM (2011). Antioxidant and cytotoxic constituents from wisteria sinensis.. Molecules..

[18] Konoshima T, Kokumai M, Kozuka M, Tokuda H, Nishino H, Iwashima A (1992). Anti-tumor-promoting activities of afromosin and soyasaponin I isolated from Wistaria brachybotrys.. J Nat Prod..

[19] Konoshima T, Kozuka M (1991). Constituents of leguminous plants, XIII. New triterpenoid saponins from Wistaria brachybotrys.. J Nat Prod..

[20] Jaya Prakkash MA, Ragunathan R, Jesteena J (2019). Evaluation of bioactive compounds from Jasminum polyanthum and its medicinal properties.. J drug deliv ther ..

[21] Singh AK (2006). Flower crops: Cultivation and management..

[22] Gregg KB, Klotz LH (2015). The flora of beavers' Meadow, Barbour County, West Virginia, revisited after a quarter century.. Castanea..

[23] Flores HE, Galston AW (1982). Analysis of polyamines in higher plants by high performance liquid chromatography.. Plant Physiol..

[24] Minocha SC, Minocha R, Robie CA (1990). High-performance liquid chromatographic method for the determination of dansyl-polyammines.. J Chromatogr A..

[25] Partridge M, Murphy DJ (2009). Roles of a membrane-bound caleosin and putative peroxygenase in biotic and abiotic stress responses in Arabidopsis.. Plant Physiol Biochem..

[26] Sanchez-Lopez J, Camanes G, Flors V, Vicent C, Pastor V, Vicedo B (2009). Underivatized polyamine analysis in plant samples by ion pair LC coupled with electrospray tandem mass spectrometry.. Plant Physiol Biochem..

[27] Magnes C, Fauland A, Gander E, Narath S, Ratzer M, Eisenberg T (2014). Polyamines in biological samples: rapid and robust quantification by solid-phase extraction online-coupled to liquid chromatography-tandem mass spectrometry.. J Chromatogr A..

[28] Janaki Ammal EK, Saunders B (1952). Chromosome numbers in species of lonicera.. Kew Bulletin..

[29] Hakkinen MR, Keinanen TA, Vepsalainen J, Khomutov AR, Alhonen L, Janne J (2008). Quantitative determination of underivatized polyamines by using isotope dilution RP-LC-ESI-MS/MS.. J Pharm Biomed Anal..

[30] Gugliucci A (2004). Polyamines as clinical laboratory tools.. Clin Chim Acta..

[31] Nasiri A, Jahani R, Mokhtari S, Yazdanpanah H, Daraei B, Faizi M (2021). Overview, consequences, and strategies for overcoming matrix effects in LC-MS analysis: a critical review .. Analyst..

[32] Jain R, Mudiam MK, Chauhan A, Ch R, Murthy RC, Khan HA (2013). Simultaneous derivatisation and preconcentration of parabens in food and other matrices by isobutyl chloroformate and dispersive liquid-liquid microextraction followed by gas chromatographic analysis.. Food Chem..

[33] Mohajeri M, Ayatollahi SA, Kobarfard F, Goli M, Khandan M, Mokhtari S Wheat germ, a byproduct of the wheat milling industry, as a good source of anti-aging polyamines: a quantitative comparison of various forms.. Food Sci Nutr ..

[34] Verdolin LG, Mariz BL, Dias LLC (2021). Gibberellin and polyamines effects in growth and flowering of New Guinea impatiens.. Ornam Hortic..

[35] Bardocz S, Grant G, Brown D, Ralph A, Pusztai A (1993). Polyamines in food—implications for growth and health.. J Nutr Biochem ..

[36] Liu JH, Moriguchi T (2007). Changes in free polyamines and gene expression during peach flower development.. Biol Plant..

[37] Azizi Gannouni T, Campoy JA, Quero-García J, Barreneche T, Arif A, Albouchi A (2017). Dormancy related traits and adaptation of sweet cherry in Northern Africa: A case of study in two Tunisian areas.. Sci Hortic ..

[38] Ding J, Liu S, Xiao HM, Ye TT, Zhou P, Feng YQ (2017). Matrix-assisted laser desorption/ionization mass spectrometry for the analysis of polyamines in plant micro-tissues using cucurbituril as a host molecule.. Anal Chim Acta..

[39] Fujihara S, Yoneyama T (2001). Endogenous levels of polyamines in the organs of cucumber plant (Cucumis sativus) and factors affecting leaf polyamine contents.. Physiol Plant..

